# Treatment status of iron medications for anemia in patients visiting gynecological or obstetric facilities: analyses using Japanese claims data

**DOI:** 10.1186/s12905-026-04769-5

**Published:** 2026-08-08

**Authors:** Jun Takeda, Tomomi Takeshima, Kosuke Iwasaki, Yohei Sonoyama, Natsumi Honda, Hiromichi Niida, Kaori Koga

**Affiliations:** 1 Department of Obstetrics and Gynecology, Juntendo University Faculty of Medicine, Tokyo, Japan; 2 Milliman, Inc, Tokyo, Japan; 3 Medical Planning Dept, Nippon Shinyaku Co.,Ltd, Kyoto, Japan; 4 Department of Obstetrics and Gynecology, Reproductive Medicine Graduate School of Medicine Chiba University, Chiba, Japan

**Keywords:** Anemia, Gynecological disorders, Intravenous iron infusions, Iron replenishment, Japan, Obstetric disorders, Oral iron medications, Pregnancy, Real-world data, Reproductive age

## Abstract

**Background:**

Anemia is highly prevalent among women of reproductive age and can have negative health effects. Thus, appropriate and effective treatment is essential to mitigate these effects. This study assessed the current treatment status of iron-deficiency anemia in this population.

**Methods:**

Data from women aged 20–49 years who visited gynecological or obstetric facilities were analyzed using a Japanese health insurance claims database (June 2018–August 2022) provided by DeSC Healthcare, Inc.; the database included health checkup data. Patients were identified based on diagnoses of gynecological or obstetric disorders, diagnoses of obstetric supervision, or family identification based on a newborn baby. The duration of oral iron administration, the amount of iron replenished by intravenous (IV) iron infusions (saccharated ferric oxide [SFO] or ferric carboxymaltose [FCM]), and the status of oral iron medication prescriptions following IV iron infusions were analyzed.

**Results:**

The median treatment durations with oral iron were 4.1 weeks for all patients, 4.4 weeks for those with Hb levels of 8–10 g/dL, and 8.7 weeks for those with Hb levels < 8 g/dL. The doses of IV iron preparations, particularly SFO, were often lower than the required doses calculated based on Hb levels and body weight. The required dose was achieved in 0.7% of the total administration episodes for SFO and in 46.4% of those for FCM. Oral iron medications were prescribed within 4 weeks after IV infusion for 37.5% of the SFO administration episodes and 20.8% of the FCM administration episodes. The iron dose administered per episode via oral medications exceeded that administered via IV infusions with SFO.

**Conclusions:**

Treatment duration of approximately 1 month with oral iron appears insufficient to fully replenish iron reserves. IV iron therapy doses were often lower than required. Despite recommendations against following IV iron therapy with oral iron treatment, frequent use of oral iron medications was observed shortly after IV infusion. Considering the potential negative effects of anemia, women of reproductive age should receive appropriate and effective treatment for iron-deficiency anemia, regardless of the presence of subjective symptoms.

**Supplementary Information:**

The online version contains supplementary material available at https://doi.org/10.1186/s12905-026-04769-5.

## Background

Anemia is highly prevalent among women of reproductive age and is often caused by abnormal uterine bleeding, pregnancy, and postpartum hemorrhage [1]. Anemia is considered a threat to women of this age in both developed and developing countries [2]. The World Health Organization includes a 50% reduction of anemia in women of reproductive age in the Global Nutrition Targets 2025 and recommends intermittent iron supplementation for menstruating women living in areas with an anemia prevalence of > 20% [3]. In Japan, a study using real-world data reported that approximately 20% of women in their 30s and 30% of women in their 40s had hemoglobin (Hb) levels < 12 g/dL [4]. From the perspective of preconception care, the issues of underweight and undernutrition among young women in Japan have also been highlighted [5–7], suggesting that the high prevalence of anemia is associated with being underweight [6].

Anemia exerts various effects on women of reproductive age. Anemia has been reported to influence some outcomes in pregnant women, such as preterm birth [8], low birth weight [8], impaired child neurodevelopment [9], postpartum depression [10], and postpartum hemorrhage [8]. The risk of pregnancy complications associated with Hb levels was also suggested [11]. Moreover, its impact on work productivity has been documented [4, 12], and it may be relevant to women in this age group. Considering these effects, appropriate anemia treatment is considered necessary.

Anemia in women of reproductive age is primarily due to iron deficiency, which may require targeted interventions. However, existing reports suggest that treatment is insufficient. A Japanese claims-based study indicated that most patients with Hb levels ≤ 12 g/dL were untreated [4]. Other Japanese claims-based studies have reported that the administered doses of intravenous (IV) iron infusions were insufficient for many patients [13, 14]. Iron-deficiency anemia often progresses slowly, leading to few subjective symptoms until the condition becomes severe [15, 16], and common anemia symptoms, such as tiredness, fatigue, dizziness, and dyspnea, may not be recognized as symptoms of a medical issue, leading individuals to refrain from reporting them to their physicians [17]. Even when treatment is initiated, it may not be appropriately administered.

Considering the high prevalence of iron-deficiency anemia, the possibility of inadequate treatment, and its effects, understanding the treatment status of iron-deficiency anemia among women of reproductive age is essential. Although some studies have reported the treatment status in Japan [4, 14], studies focusing on women of reproductive age are scarce. This study aims to assess the current treatment status of iron-deficiency anemia, focusing on the duration of oral iron administration, the amount of iron replenished by IV iron infusions, and the status of oral iron prescriptions following IV iron infusions. In particular, individuals who visited gynecological or obstetric facilities were targeted.

## Methods

### Study design and data sources

This claims-based study assessed the current treatment status with iron medications. The Japanese health insurance claims database provided by DeSC Healthcare, Inc. (Tokyo, Japan) was used, covering June 2018 to August 2022. The database consists of enrollee data from various insurers, including national health insurance, health insurance societies, and the medical care system for the advanced elderly in Japan. It included data from approximately 2.5 million women aged ≤ 64 years. It also contained health checkup examination data from a subset of individuals, and information on the Hb value and body weight was obtained from the data.

### Subjects

The subjects of this study were women aged 20–49 years who visited gynecological or obstetric facilities. These subjects were extracted from the database according to the following criteria: women within this age range who had a definitive diagnosis of any gynecological disorders, obstetric disorders, or obstetric supervision (hereafter referred to as “gynecological or obstetric-related diagnosis”) or who were pregnant. The diagnoses were based on the International Statistical Classification of Diseases and Related Health Problems, 10th Revision codes: D25 (leiomyoma of uterus), D26 (other benign neoplasms of uterus), E28 (ovarian dysfunction), N70–N77 (inflammatory diseases of female pelvic organs), and N80–N98 (noninflammatory disorders of female genital tract) for gynecological disorders and O10–O99 (pregnancy, childbirth and the puerperium excluding pregnancy with abortive outcome), Z33 (pregnant state, incidental), Z34 (supervision of normal pregnancy), and Z35 (supervision of high-risk pregnancy) for obstetric disorders or supervision. Pregnant women were identified by having the same family ID as the insured child, with the pregnancy period defined as the 10 months prior to the child’s birth month. Pregnancy and childbirth-related expenses are covered in other ways in Japan [18]; therefore, not all pregnant women were defined based on the claims data.

### Iron medications

The treatment status of iron medications was examined for oral and IV administrations (Table A1 in Additional File 1). Saccharated ferric oxide (SFO) and ferric carboxymaltose (FCM) were the only types of IV iron infusions available in Japan during the study period and were separately analyzed.

An episode of treatment was defined as a series of consecutive prescriptions. For oral iron medications, consecutive prescriptions were defined as those in which the interval between the previous prescription date plus the number of days prescribed and the next prescription date was < 14 days. The start date of the episode was the earliest prescription date among the consecutive prescriptions, and the end date of the episode was the latest prescription date plus the number of days prescribed. The duration of the episode was defined as the period from the start date of the episode to the end date of the episode. The duration of the administration period was defined as the duration of the episode.

For IV iron infusions, consecutive administrations were defined as those in which the interval between the previous and next administration dates was < 28 days. The required dose was estimated based on the “total iron dose” or “total dose” calculation methods described in the package inserts (Table A2 in Additional File 1) [19, 20] using the Hb level and body weight. The latest Hb value and body weight before the start of the episode were used. The actual dose was defined as the total dose administered during the episode.

### Statistical analysis

We utilized descriptive statistics in this study.

The distribution of individuals for each treatment combination (oral iron, SFO, FCM, and blood transfusion [Table A1 in Additional File 1]) in September 2020–August 2022 was tabulated according to the following Hb levels: <8, 8–<10 (8–10), 10–<12 (10–12), and ≥ 12 g/dL. This period was chosen because it corresponds to the time after SFO and FCM became available for clinical use. In this analysis, women aged 20–49 years as of August 2021 with an observation period (defined as the period included in the database for each individual) between September 2020 and August 2022 had at least one health checkup data of Hb level during the period and met any of the following conditions were included: having a gynecological or obstetric-related diagnosis during the study period or having the same family ID as insured individuals whose birth month was in September 2020 or later. Hb levels during the study period were used, and the lowest value was used when several data points were available.

The background characteristics and medical setting of patients who were prescribed iron medications were examined according to treatment type. The number of patients was tabulated according to their background characteristics and medical setting according to medication type. The background and medical setting included age at the index (defined as the first date of prescription), latest Hb level before the index, inpatient or outpatient status as recorded on the provisional claim of iron prescription at the index, and type of diagnosis (gynecological or obstetric) recorded on the provisional claim of iron prescription at the index. In this analysis, women aged 20–49 years during the observation period who had a prescription of SFO, FCM, or oral iron as either one at the index, had an observation period that ended later than the month of the index date + 8 weeks, had Hb level data within 3 months before the index, and had a gynecological or obstetric-related diagnosis as recorded on the provisional claim of iron prescription at the index were analyzed.

The administration period per episode of oral iron medication was examined for all patients as well as for patients grouped according to the following Hb levels: <8, 8–10, 10–12, and ≥ 12 g/dL. Patients included in this study had prescriptions for oral iron medications and episodes that met all the following criteria. A single individual can have multiple episodes. The criteria were as follows:

- The age at the start of the episode was between 20 and 49 years.
- An Hb value was available within 3 months before the start of the episode.
- The episode occurred within the observation period, with the month in which the episode started being after the month following the start of the observation period and the month in which the episode ended being before the month of the end of the observation period.
- At the start of the episode, either a gynecological or obstetric-related diagnosis was recorded, or pregnancy was determined.

The administration status of IV iron infusion was examined by calculating the iron dose administered in each episode and comparing it with the required dose. The episodes were examined separately for SFO and FCM, and any episodes that included both SFO and FCM were excluded from the analysis. The number of injections per episode was also investigated for SFO/FCM. The target patients were those who had prescriptions for IV iron infusions and had episodes that met all of the following criteria. A single individual can have multiple episodes.

- The age at the start of the episode was between 20 and 49 years.
- Hb and body weight data were available within 3 months before the start of the episode.
- The episode occurred within the observation period, with the month in which the episode started being after the second month following the start of the observation period and the month in which the episode ended being before the month prior to the end of the observation period.
- At the start of the episode, either a gynecological or obstetric-related diagnosis was recorded, or pregnancy was determined.

The treatment status with oral iron after IV iron administration was investigated. The episodes for SFO and FCM were examined separately. For this analysis, patients prescribed IV iron infusions and had episodes that met all of the following criteria were included. A single individual can have multiple episodes. The criteria were as follows:

- The age at the start of the episode was between 20 and 49 years.
- The episode occurred within the observation period, with the month in which the episode started being after the second month following the start of the observation period and the month in which the episode ended being before the month prior to the end of the observation period, and the month in which the episode started plus 4 weeks being before the month of the end of the observation period.

The number of episodes in which oral iron was prescribed within 4 weeks from the start of the SFO/FCM episode and the iron content administered per episode *via* IV or oral iron were calculated. The amount of iron *via* IV iron infusions was calculated by dividing the total amount of IV iron infusions prescribed during the SFO/FCM episode period by the number of episodes. The amount of iron administered *via* oral medications was calculated by dividing the total amount of iron (iron content × number of prescription days × number of prescriptions) of all oral iron medications prescribed during the SFO/FCM episode period by the number of episodes.

## Results

In total, 23,480 individuals were extracted as target patients who met the criteria to observe the distribution of treatment combinations in September 2020–August 2022. Among individuals with Hb levels of 10–12 g/dL, 60.4% did not receive any treatment (Table A3 in Additional File 1). Among individuals with Hb levels of 8–10 g/dL, 23.8% did not receive any treatment, and 61.6% received only oral iron. Among those with Hb levels < 8 g/dL, 9.8% received no treatment, and 51.8% received only oral iron.

As described in Table 1, 425 patients prescribed iron medications were selected to analyze of background characteristics and medical settings. For all attribute groups, oral treatment was more common than IV infusion (Table 2). Regardless of medication type, a higher percentage of individuals received treatment in outpatient settings than in inpatient settings.

**Table 1:** Selection (Table 1) and background (Table 2) of patients with gynecological/obstetric disorders who were prescribed iron treatments. The number of individuals remaining after each selection criterion is applied, showing the step-by-step selection process. Each number represents the remaining individuals after applying the criterion in that row to the previously filtered group

Criteria	Selected individuals, *N*
Number of individuals in the dataset: women aged 64 years	2,507,837
Those who had at least one health checkup data of Hb level during the study period and aged 20–49 years during the observation period	140,925
Those with a prescription of SFO, FCM, or oral iron	21,433
Those whose observation period ended later than the month of the index date + 8 weeks	20,294
Those with Hb level data within 3 months before the index	4392
Those with a definitive diagnosis of any gynecological disorders, obstetric disorders, or obstetric supervision, recorded on the provisional claim of iron prescription at the index	425

*FCM* ferric carboxymaltose, *Hb* hemoglobin, *IV* intravenous, *N* number, *SFO * saccharated ferric oxide

**Table 2:** Selection (Table 1) and background (Table 2) of patients with gynecological/obstetric disorders who were prescribed iron treatments. The distribution of selected patients according to background characteristics and medical setting according to treatment type

		Patients prescribed each treatment, N		
		Oral drug	IV infusion	
			SFO	FCM
Age (years)	20–29	81	2	0
	30–39	112	7	2
	40–49	182	35	4
Hb level (g/dL)	< 8	20	10	4
	8–10	87	16	1
	10–12	208	11	0
	≥ 12	60	7	1
Inpatient/outpatient	Inpatient	62	1	0
	Outpatient	313	43	6
Type of diagnosis	Gynecological	248	42	5
	Obstetric	127	2	1

*FCM* ferric carboxymaltose, *Hb* hemoglobin, *IV* intravenous, *N* number, *SFO * saccharated ferric oxide

Table 3 presents the distribution of the duration of oral iron administration per episode. The period was longer for patients with lower Hb levels; the median periods were 4.1 weeks for all individuals, 4.4 weeks for those with Hb levels of 8–10 g/dL, and 8.7 weeks for those with Hb levels < 8 g/dL.

**Table 3:** Duration of oral iron administration per episode

	Hb level (g/dL)			
	< 8	8–10	10–12	≥ 12
Minimum	0.9	1.6	0.4	0.6
25%ile	5.1	4.1	2.1	1.6
50%ile	8.7	4.4	3.1	2.8
75%ile	13.4	8.6	4.4	4.4
Maximum	35.6	63.7	40.1	20.1

The number in each column represents the duration of the administration period per episode in weeks

*Abbreviation Hb *hemoglobin

**Fig. 1:**
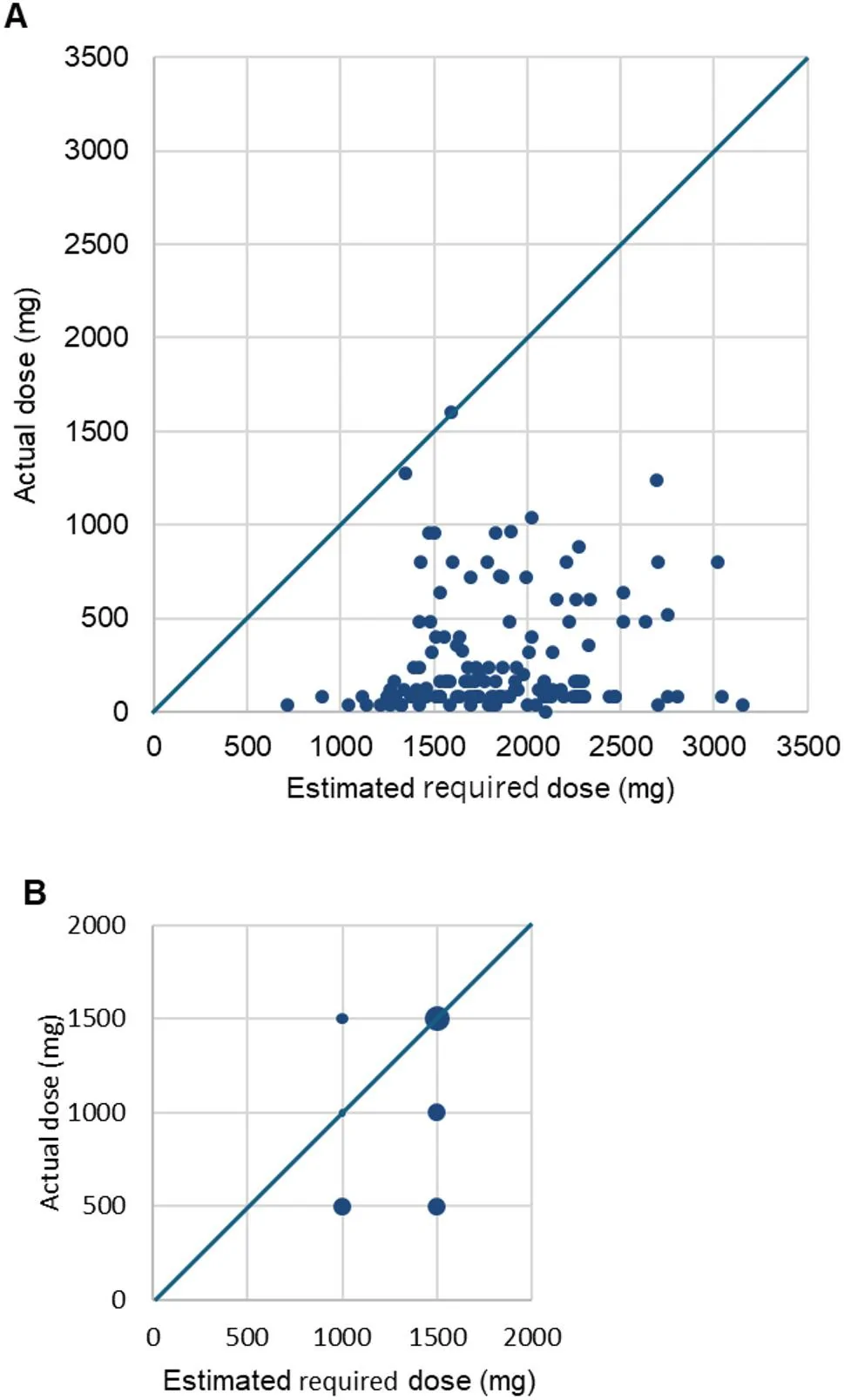
Actual and required iron doses per episode for SFO (**A**) or FCM (**B**). The total number of episodes included in this analysis was 147 for SFO and 28 for FCM. One circle represents each episode for (**A**), and the size of the circle represents the number of episodes for (**B**). Abbreviations: SFO, saccharated ferric oxide; FCM, ferric carboxymaltose

Regarding IV iron infusion, the actual iron dose administered was lower than the estimated required dose (Fig. 1). Episodes that reached the estimated required dose were 0.7% for SFO and 46.4% for FCM. For SFO and FCM, one injection per episode was the most common, accounting for 46.9% and 35.7% of the episodes, respectively (Fig. A1 in Additional File 1).

In 37.5% of the SFO administration episodes and 20.8% of the FCM administration episodes, oral iron medications were prescribed within 4 weeks after the start of the episode (Table 4). For SFO, the iron dose administered per episode *via* oral medications exceeded that administered *via* IV infusions.

**Table 4:** Prescription of oral iron medications after the IV iron administration episode started

Type	*N* of episodes	*N* (%) of episodes prescribed oral iron within 4 weeks after the IV infusion episode started	Amount of iron per episode via IV infusions (mg)	Amount of iron per episode via oral medications (mg)
SFO	5598	2100 (37.5%)	276	733
FCM	370	77 (20.8%)	954	224

*Abbreviations* *FCM * ferric carboxymaltose, *IV * intravenous, *N * number, *SFO * saccharated ferric oxide

## Discussion

In this study, we analyzed health insurance claims data to examine the treatment status of iron medications for iron-deficiency anemia in women of reproductive age who visited gynecological or obstetric facilities in Japan. Considering the high prevalence of iron-deficiency anemia and the potential negative effects of inadequate treatment on women’s health, understanding the treatment status is essential. Although some studies have reported the treatment status of anemia in Japan [4, 14] and other countries [21], studies focusing on this population are limited. Our analysis suggested that many individuals with low Hb levels were untreated and that most treated patients received only oral iron medication, including those with Hb levels < 8 g/dL. These findings were consistent with those described in a study targeting the entire Japanese population [4]. Among patients treated with oral iron medication, the median treatment duration was approximately 1 month. The doses of the IV iron preparations, particularly SFO, were often lower than required, and frequent use of oral iron medications was observed shortly after IV infusion.

We found that the treatment duration with oral iron medication was approximately 1 month for many patients. Treatment may be discontinued if the Hb levels improve or symptoms are alleviated. However, although Hb levels typically normalize within 6–8 weeks, serum ferritin levels gradually increase following the increase in Hb [15]. Therefore, iron treatment should continue until Hb and serum ferritin levels have normalized. The optimal response to oral iron medication is generally defined as an increase of 2 g/dL in Hb levels after 3 weeks; however, the ultimate goal should be the replenishment of iron stores, which often requires long-term treatment of at least 3 months [22]. Especially for pregnant women, the Guidelines for obstetrical practice in Japan recommend a treatment duration of at least 6 weeks for those with anemia who are undergoing iron supplementation [23]. Considering these reports and recommendations, it is possible that many patients receiving oral iron supplements are not undergoing sufficient treatment to fully address iron deficiency. Adequate treatment of anemia should be administered to women of reproductive age, even in the absence of subjective symptoms. Particularly, considering that pregnancy usually involves regular hospital visits and it will often be the first one for them, it presents a crucial opportunity to screen and treat anemia.

Our study indicated that the actual administered doses of IV iron therapy were often lower than the required doses, which are calculated based on the patient’s Hb level and body weight prior to administration. Additionally, single-dose administration was often observed. These results suggest that IV iron therapy is often administered without the physician being aware of the required dose. If the administered iron dose is insufficient, it is likely that the need for retreatment increases or there is a subtherapeutic response risk [24, 25]. Therefore, educating physicians on administrating appropriate IV iron doses to effectively manage iron-deficiency anemia appears necessary.

We found that oral iron was administered after IV iron administration, particularly in patients receiving SFO. Notably, IV iron therapy is considered for patients who require rapid iron repletion or those who are poor responders to oral iron medications, particularly when Hb levels do not increase by 1 g/dL after 2–3 weeks of supplementation [26]. However, oral iron treatment after IV iron therapy is generally not recommended. The primary reason is that IV iron therapy increases serum hepcidin levels [27], which impairs iron absorption due to hepcidin’s role in inhibiting iron uptakes [16, 28].

This study provides a better understanding of anemia treatment in women of reproductive age by analyzing insurance claims data from gynecology and obstetrics patients. However, several limitations should be considered when interpreting the results. First, the distribution of insurance types and other demographic attributes in the database may not be consistent with the Japanese population. Moreover, focusing on women who visited gynecological or obstetric facilities led to a small sample size, which may have affected the generalizability of the findings. Furthermore, individuals with health checkup data included in the analyses using Hb values may have a higher level of health consciousness than average individuals. Second, Hb values from health checkup data, not for diagnosing or treating anemia, were used to calculate the required iron dose. Although the values within 3 months before the treatment episode were used, Hb levels may have improved after measurement due to treatment outside the facility where the episode was observed. In such cases, the required dose calculated using data at the time of diagnosing or treating anemia may be lower than that calculated based on the health checkup data, and the treatment may be considered insufficient. Third, normal deliveries are not covered by health insurance and thus not recorded in the database. Consequently, pregnant women were identified using family IDs, including only cases in which both the mother and child were insured together, potentially underestimating the number of pregnant women. Moreover, the pregnancy period was defined as 10 months preceding the child’s birth date, which may not accurately reflect the actual pregnancy duration. Fourth, the presence of treatment was determined based on the prescriptions for iron medications and blood transfusions. However, patients with anemia types other than iron-deficiency anemia, such as hemolytic and renal anemia, who received appropriate treatments other than iron supplementation were still classified as untreated. Nevertheless, most patients with low Hb levels who visit gynecological and obstetric clinics are more likely to have iron-deficiency anemia rather than other types of anemia. Finally, over-the-counter (OTC) medications are available for treating iron-deficiency anemia; however, such treatments are not captured by insurance claims data. Therefore, some individuals classified as untreated may be receiving OTC treatments. Nevertheless, because the study population comprised patients visiting medical facilities, those experiencing subjective symptoms due to anemia were likely to prefer prescriptions from the medical facilities over OTC options. Although the data covered 2018–2022, they provide an important benchmark of real-world treatment patterns immediately before and during the early period following the introduction of ferric carboxymaltose in Japan. To our knowledge, nationwide evidence describing treatment duration of oral iron therapy, dosing of intravenous iron, and prescribing patterns after intravenous iron therapy in women of reproductive age remains limited.

## Conclusions

Our study demonstrated that in Japan, treatment for iron-deficiency anemia in women who visited gynecological or obstetric facilities was often not appropriate, including short duration of oral treatment, insufficient doses of IV iron treatments, and frequent prescription of oral iron medications following IV treatment. Based on these findings regarding the treatment practices at Japanese medical facilities, it is essential to standardize appropriate treatment for iron-deficiency anemia. To achieve this, some measures, including physician education on the proper treatment with iron medications and providing insurance incentives, are considered necessary.

## Supplementary Information

Additional file 1.

## Data Availability

The data that support the findings of this study are available from DeSC Healthcare, Inc., but restrictions apply to the availability of these data, which were used under license for the current study, and so are not publicly available. Data are, however, available from the authors upon reasonable request and with permission of DeSC Healthcare, Inc.

## Abbreviations

FCM: Ferric carboxymaltose
Hb: Hemoglobin
IV: Intravenous
OTC: Over-the-counter
SFO: Saccharated ferric oxide
