# Supplementary material for: Treatment status of iron medications for anemia in patients visiting gynecological or obstetric facilities: analyses using Japanese claims data
Source: BMC Womens Health. 2026 Aug 8;26:586. doi: 10.1186/s12905-026-04769-5 (PMC13732869; doi:10.1186/s12905-026-04769-5)
Supplement: Supplementary file 1 [file 12905_2026_4769_MOESM1_ESM.docx]

Table A1. Definition of treatment for iron-deficiency anemia

| Type of treatment | Definition (receipt computer processing system code) |
| --- | --- |
| IV iron infusion | Saccharated ferric oxide (Drug code: 620005208) |
|  | Ferric carboxymaltose (Drug code: 622676601) |
| Oral iron | Ferrous fumarate (Drug code: 620729301)  Sodium ferrous citrate (Drug code: 622738100, 621307601, 620006090, 620730201, 620730403, 620730404, 621307701, 622738200, 622288601, 613220039, 610407094, 620007053)  Ferrous sulfate (Drug code: 620006032, 620729101)  Ferric citrate hydrate (Drug code: 622306901) |
| Blood transfusion | Transfusion management fee (Procedure code: 150327910, 150328010) |

Table A2. Total iron dose required (mg) according to baseline body weight and baseline hemoglobin concentration, based on the approved package inserts for saccharated ferric oxide (A) and ferric carboxymaltose (B).

A.

|  | | **Baseline hemoglobin concentration (g/dL)** | | | | | | | | |
| --- | --- | --- | --- | --- | --- | --- | --- | --- | --- | --- |
|  |  | 5 | 6 | 7 | 8 | 9 | 10 | 11 | 12 | 13 |
| **Baseline body weight (kg)** | 20 | 940 | 880 | 830 | 780 | 720 | 670 | 610 | 560 | 500 |
|  | 30 | 1410 | 1330 | 1240 | 1160 | 1080 | 1000 | 920 | 840 | 750 |
|  | 40 | 1880 | 1770 | 1660 | 1550 | 1440 | 1330 | 1220 | 1120 | 1010 |
|  | 50 | 2350 | 2210 | 2070 | 1940 | 1800 | 1670 | 1530 | 1390 | 1260 |
|  | 60 | 2820 | 2650 | 2490 | 2330 | 2160 | 2000 | 1840 | 1670 | 1510 |
|  | 70 | 3280 | 3090 | 2900 | 2710 | 2520 | 2330 | 2140 | 1950 | 1760 |

B.

|  | | **Baseline hemoglobin concentration (g/dL)** | |
| --- | --- | --- | --- |
|  |  | **<10.0** | **≥10.0** |
| **Baseline body weight category (kg)** | 25–<35 | 500 | 500 |
|  | 35–<70 | 1500 | 1000 |
|  | ≥70 | 1500 | 1500 |

The value at the intersection of body weight and hemoglobin category represents the cumulative elemental iron dose recommended for the treatment of iron-deficiency anemia. How to read this table: select the patient’s baseline body weight or body weight category (row) and baseline hemoglobin concentration or hemoglobin category before treatment (column). The number shown at the intersection indicates the total iron dose (mg) recommended according to the package insert. For panel A, the required dose for saccharated ferric oxide is determined by both body weight and hemoglobin level. For panel B, the required dose for ferric carboxymaltose is determined according to body weight category and whether hemoglobin is below 10.0 g/dL or 10.0 g/dL or higher.

References: A. Fesin injection 40mg. Package insert. Nichi-iko Pharmaceutical Co., Ltd.; 2023 [cited January 24, 2025]. Available from: https://www.info.pmda.go.jp/go/pack/3222400A1058_1_04/ (Japanese); B. Ferinject solution for injection/infusion 500mg. Package insert. Zeria Pharmaceutical Co., Ltd.; 2021 [cited January 24, 2025]. Available from: https://www.info.pmda.go.jp/go/pack/3222404A1021_1_04/ (Japanese).

Table A3. Distribution of individuals according to a combination of treatment types within each hemoglobin level

| Type  of  treatment | Oral iron drug | |  | - | + | - | - | - | + | - | - | + | + | - | + | - | + | + | + |
| --- | --- | --- | --- | --- | --- | --- | --- | --- | --- | --- | --- | --- | --- | --- | --- | --- | --- | --- | --- |
|  | IV　iron  infusion | SFO |  | - | - | + | - | - | + | + | - | - | - | + | + | **+** | + | - | + |
|  |  | FCM |  | - | - | - | + | - | - | + | + | - | + | - | + | + | - | + | + |
|  | Blood transfusion | |  | - | - | - | - | + | - | - | + | + | - | + | - | + | + | + | + |
|  | | | N | Distribution within the Hb level (%) | | | | | | | | | | | | | | | |
| Hb level  (g/dL) | <8 | | 164 | 9.8% | 51.8% | 4.3% | 4.9% | 1.2% | 17.7% | 1.2% | 0.0% | 1.2% | 4.3% | 0.0% | 3.0% | 0.0% | 0.0% | 0.6% | 0.0% |
|  | 8 to <10 | | 984 | 23.8% | 61.6% | 2.7% | 1.2% | 0.3% | 6.9% | 0.8% | 0.0% | 0.5% | 1.1% | 0.0% | 0.6% | 0.0% | 0.2% | 0.1% | 0.1% |
|  | 10 to 12 | | 4379 | 60.4% | 32.5% | 1.3% | 0.3% | 0.1% | 4.0% | 0.1% | 0.0% | 0.3% | 0.4% | 0.0% | 0.3% | 0.0% | 0.2% | 0.0% | 0.0% |
|  | ≥12 | | 17953 | 87.7% | 9.6% | 0.6% | 0.1% | 0.1% | 1.3% | 0.0% | 0.0% | 0.2% | 0.2% | 0.0% | 0.1% | 0.0% | 0.1% | 0.0% | 0.0% |

N represents the number of individuals according to Hb level. The percentage in each column indicates the percentage of individuals who received each treatment indicated as “+” in the above lines any time between September 2020 and August 2022 within each hemoglobin level.

Abbreviations: FCM, ferric carboxymaltose; Hb, hemoglobin; IV, intravenous; SFO, saccharated ferric oxide.

Figure A1. Distribution of episodes according to the number of injections per episode for SFO (A) or FCM (B)

**A**

Distribution of episodes

| Number of injections | ％ |
| --- | --- |
| 1 | 46.9388% |
| 2 | 14.2857% |
| 3 | 8.16327% |
| 4 | 4.7619% |
| 5 | 6.12245% |
| 6 | 2.72109% |
| 7 | 0.680272% |
| 8 | 2.04082% |
| 9 | 2.72109% |
| 10 | 4.7619% |
| 11 | 1.36054% |
| 12 | 2.04082% |
| 13 | 0.680272% |
| 14 | 0% |
| 15 | 0% |
| 16 | 1.36054% |
| 17 | 0% |
| 18 | 0% |
| 19 | 0% |
| 20 | 0.680272% |
| 21 | 0% |
| 22 | 0% |
| 23 | 0% |
| 24 | 0% |
| 25 | 0% |
| 26 | 0% |
| 27 | 0% |
| 28 | 0% |
| 29 | 0% |
| 30 | 0% |
| 31 | 0% |
| 32 | 0.680272% |

**B**

Destribution of episodes

| Number of injections | ％ |
| --- | --- |
| 1 | 35.7143% |
| 2 | 21.4286% |
| 3 | 42.8571% |

The total number of episodes included in this analysis was 147 for SFO and 28 for FCM.

Abbreviations: SFO, saccharated ferric oxide; FCM, ferric carboxymaltose.

Figure A2. Schematic representation of observation periods and eligibility windows (A) Overall study framework; (B) For background/medical setting; (C) For oral iron episode analysis; (D) For IV iron (SFO/FCM) episode analysis; and (E) For oral iron after IV iron analysis. The observational period represents the period during which claims data were available for each individual in the database. Shaded areas highlight analysis-specific windows used to ensure the availability of necessary information before and after each episode.
